# Supplementary material for: Effect of Hermetia illucens Fat, Compared with That of Soybean Oil and Palm Oil, on Hepatic Lipid Metabolism and Plasma Metabolome in Healthy Rats
Source: Animals (Basel). 2023 Oct 29;13(21):3356. doi: 10.3390/ani13213356 (PMC10649396; doi:10.3390/ani13213356)
Supplement: Supplementary file 1 [file animals-13-03356-s001.zip › animals-2634580-supplementary.pdf]

Supplementary file

Table S1 Performance characteristics of gene-specific primers used for qPCR.

Gene symbol	Primer forward (von 5` nach 3`)<br>Primer reverse (von 5` nach 3`)	PCR product size (bp)	NCBI GeneBank
Reference genes			
Canx	CCAGATGCAGATCTGAAGAC<br>CTGGGTCCTCAATTTCACGT	175	NM_172008
Mdh1	CAGACAAAGAAGAGGTTGCC<br>CGTCAGGCAGTTTGTATTGG	206	NM_033235
Rpl13	CTTAAATTGGCCACGCAGCT<br>CTTCTCAACGTCTTGCTCTG	198	NM_031101
Sdha	GCCTCCGTGGTTGAGCTAGAA<br>CGACACAGCAACACCGATGG	136	NM_130428
Target genes			
Acaca	TACAACGCAGGCATCAGAAG<br>TGTGCTGCAGGAAGATTGAC	244	NM_022193
Acly	TCAGTCCCAAGTCCAAGATCCC<br>ACGGGTAGACCATAGCAGCC	166	NM_016987
Acox1	CTCAGCAGGAGAAATGGATGCG<br>TCCTTGAGTGATGAGCTGAGCC	239	NM_017340
Cpt1a	ACCGCCATCTCTTCTGCCTC<br>CTGCTGAGGAGTCTGGCTCG	116	NM_031559
Crp	AGCTTTGGCTTGACGGGAAAC<br>CTCCCACCAGACTGATTCGC	139	NM_017096
Elovl5	ATGAACTGGGTTCCCTGCGG<br>GGAAGGGACAGAGGACAGGC	102	NM_134382

Gpam	CAGCGTGATTGCTACCTGAA	194	NM_017274
	CTCTCCGTCCTGGTGAGAAG		
G6pd	TTGTACCAGGGTGATGCCTTCC	199	NM_017006
	GCTCACTCTGTTTGCGGATGTC		
Hmgcr	TGGCAGGACGCAACCTCTAC	173	NM_013134
	GGCAGCAGGTTTCTTGTCGG		
Icam1	CTCCAATGGCTTCAACCCGTG	93	NM_012967
	ATACCTGAGCACCGACAGGC		
Il1b	AGTTGACTTCACCATGGAACCCG	298	NM_031512
	ACAATGCTGCCTCGTGACCC		
Ldlr	ACAGTGTCCCAAGTCCAA	222	NM_175762
	GCAAATGTGGATCTCGTCCTC		
Scd1	GTACTACAAGCCTGGCCTC	227	NM_139192
	CACCCAGGGAAACCAGGAT		
Tnf	CCACCAAGCGGAGGAGCAGC	187	NM_012675
	TCGGCTGACGGTGTGGGTGA		

Table S2 Concentrations of altered plasma metabolites in lean Zucker rats fed semi-synthetic diets with either soybean oil (SO), palm oil (PO) or *Hermetia* fat (HF) as the main fat source for 4 weeks.

Class	Metabolite	SO	PO	HF	*P*-value	FDR
Fatty Acids	FA(12:0)	<LOD c	42.8 ± 15.8 b	62.9 ± 16.4 a	3.90E-19	7.61E-17
Triacylglycerols	TG(18:3 36:4)	9.22 ± 2.27 a	0.532 ± 0.182 c	0.866 ± 0.293 b	2.16E-18	3.17E-16
Triacylglycerols	TG(18:2 36:5)	16.5 ± 4.3 a	0.959 ± 0.401 c	1.9 ± 0.61 b	5.78E-17	5.63E-15
Triacylglycerols	TG(18:2 36:4)	83.4 ± 19.5 a	6.73 ± 3.27 c	11 ± 3.3 b	5.23E-16	4.37E-14
Triacylglycerols	TG(18:0 30:0)	<LOD c	0.794 ± 0.487 b	1.18 ± 0.42 a	5.80E-15	4.24E-13
Triacylglycerols	TG(18:1 26:0)	0.316 c	1.35 ± 1.16 b	7.64 ± 3.52 a	3.68E-14	2.16E-12
Diacylglycerols	DG(18:2 18:2)	11.8 ± 2.3 a	2.05 ± 0.72 c	3.34 ± 0.77 b	4.65E-14	2.27E-12
Triacylglycerols	TG(22:2 32:4)	0.0817 ± 0.0171 a	0.0121 ± 0.0057 c	0.0158 ± 0.0043 b	4.64E-14	2.27E-12
Triacylglycerols	TG(20:4 36:5)	3.42 ± 1.2 a	0.275 ± 0.15 c	0.615 ± 0.166 b	8.94E-14	4.02E-12
PC	PC ae C30:0	0.121 ± 0.014 c	0.158 ± 0.017 b	0.267 ± 0.036 a	1.07E-13	4.22E-12
Triacylglycerols	TG(16:0 30:2)	0.439 ± 0.234 c	4.87 ± 3.57 b	14.9 ± 5.9 a	1.08E-13	4.22E-12
Triacylglycerols	TG(18:2 28:0)	0.335 ± 0.13 c	3.76 ± 3.14 b	14.4 ± 6 a	1.45E-13	5.30E-12
PC	PC aa C30:0	0.979 ± 0.131 c	1.48 ± 0.27 b	3.03 ± 0.72 a	6.62E-13	2.28E-11
Triacylglycerols	TG(18:0 30:1)	0.203 ± 0.042 c	1.22 ± 0.89 b	2.3 ± 0.91 a	7.15E-13	2.32E-11
Triacylglycerols	TG(16:0 28:1)	0.352 ± 0.054 c	1.77 ± 1.28 b	6.05 ± 3.07 a	1.58E-12	4.86E-11
Cholesterol Esters	CE(14:0)	3.58 ± 0.86 c	5.5 ± 1.68 b	13.5 ± 2.3 a	1.93E-12	5.64E-11
Triacylglycerols	TG(16:1 28:0)	0.238 ± 0.024 c	1.09 ± 0.98 b	5.67 ± 2.51 a	2.76E-12	7.68E-11
Triacylglycerols	TG(20:4 36:4)	17.6 ± 5.8 a	1.97 ± 1.07 c	3.6 ± 1.25 b	5.54E-12	1.47E-10
Triacylglycerols	TG(20:4 30:0)	0.421 ± 0.181 c	1.04 ± 0.52 b	3.37 ± 0.88 a	7.57E-12	1.93E-10
Triacylglycerols	TG(20:5 36:3)	4.49 ± 1.78 a	0.66 ± 0.262 c	1.28 ± 0.33 b	1.88E-11	4.58E-10
Triacylglycerols	TG(18:1 30:1)	1.32 ± 0.86 b	18.3 ± 12.2 a	28.3 ± 16.4 a	1.96E-11	4.58E-10
Triacylglycerols	TG(18:1 28:1)	0.248 ± 0.069 c	1.52 ± 0.98 b	4.39 ± 2.36 a	2.83E-11	6.14E-10
Triacylglycerols	TG(20:3 36:5)	0.214 ± 0.066 a	<LOD b	0.13 ± 0.011 b	2.78E-11	6.14E-10
Triacylglycerols	TG(17:0 32:1)	0.336 c	0.489 ± 0.208 b	0.775 ± 0.37 a	3.24E-11	6.77E-10
Triacylglycerols	TG(18:3 36:3)	14.3 ± 4.1 a	2.21 ± 0.9 c	3.44 ± 1.79 b	3.75E-11	7.56E-10
LysoPC	lysoPC a C20:3	3.21 ± 0.44 b	9.2 ± 2.41 a	9.11 ± 2.72 a	5.15E-11	1.00E-09
PC	PC ae C42:1	0.808 ± 0.132 a	0.362 ± 0.051 c	0.441 ± 0.084 b	7.87E-11	1.49E-09

Triacylglycerols	TG(20:2 36:5)	0.302 ± 0.087 a	0.0614 ± 0.0244 c	0.131 ± 0.044 b	8.26E-11	1.51E-09
Triacylglycerols	TG(18:2 30:1)	1.12 ± 0.61 c	4.95 ± 3.1 b	13.1 ± 5.6 a	9.85E-11	1.75E-09
Triacylglycerols	TG(18:1 30:2)	0.509 ± 0.154 c	3.64 ± 2.59 b	10.5 ± 4.5 a	1.21E-10	2.02E-09
PC	PC aa C34:1	38.6 ± 8 b	98.3 ± 15.9 a	85.4 ± 24.7 a	2.65E-10	4.30E-09
PC	PC aa C32:1	4.72 ± 1.54 c	13.2 ± 4.1 b	19.4 ± 7 a	3.30E-10	5.21E-09
Triacylglycerols	TG(18:0 36:5)	2.15 ± 0.64 a	0.424 ± 0.166 c	0.671 ± 0.232 b	3.59E-10	5.52E-09
Triacylglycerols	TG(18:1 36:5)	13 ± 3.7 a	2.76 ± 1.02 c	4.79 ± 1.98 b	3.99E-10	5.99E-09
Triacylglycerols	TG(18:2 38:6)	9.9 ± 3.35 a	1.61 ± 0.85 c	3.61 ± 1 b	5.91E-10	8.65E-09
Triacylglycerols	TG(18:2 34:4)	2.32 ± 0.89 a	0.501 ± 0.171 c	1.24 ± 0.43 b	7.22E-10	1.03E-08
Triacylglycerols	TG(20:3 32:0)	0.36 ± 0.126 b	1.71 ± 0.88 a	2 ± 0.95 a	8.44E-10	1.18E-08
Triacylglycerols	TG(17:0 36:4)	1.19 ± 0.29 a	0.297 ± 0.142 c	0.511 ± 0.176 b	1.05E-09	1.43E-08
PC	PC aa C28:1	0.337 ± 0.053 c	0.452 ± 0.057 b	0.649 ± 0.099 a	1.28E-09	1.70E-08
Triacylglycerols	TG(16:1 30:1)	0.685 ± 0.304 c	2.8 ± 2.4 b	7.12 ± 4.22 a	1.53E-09	1.95E-08
Triacylglycerols	TG(18:1 30:0)	2.1 ± 1.43 b	19.1 ± 15.4 a	23.4 ± 12.2 a	1.53E-09	1.95E-08
Fatty Acids	EPA	1.28 ± 0.52 a	0.34 ± 0.107 b	0.439 ± 0.087 b	1.71E-09	2.11E-08
Triacylglycerols	TG(20:3 32:1)	0.579 ± 0.347 c	2.48 ± 1.22 b	4.19 ± 1.97 a	1.73E-09	2.11E-08
Triacylglycerols	TG(18:2 35:3)	0.853 ± 0.198 a	0.21 ± 0.09 c	0.387 ± 0.135 b	1.79E-09	2.13E-08
Triacylglycerols	TG(22:5 32:1)	1.05 ± 0.54 c	2.46 ± 0.83 b	5.75 ± 2.04 a	2.77E-09	3.24E-08
Triacylglycerols	TG(20:3 34:1)	3.02 ± 1.54 b	13 ± 5.7 a	17.6 ± 8.7 a	2.97E-09	3.41E-08
Triacylglycerols	TG(18:2 33:2)	2.6 ± 0.72 a	0.672 ± 0.33 c	1.24 ± 0.38 b	4.17E-09	4.61E-08
Triacylglycerols	TG(14:0 36:1)	0.606 ± 0.231 b	3.39 ± 2.43 a	4.36 ± 2.11 a	4.35E-09	4.72E-08
Triacylglycerols	TG(14:0 36:2)	3.28 ± 2.22 b	21.5 ± 15.3 a	29.3 ± 15.4 a	4.54E-09	4.74E-08
Triacylglycerols	TG(18:1 34:1)	87.2 ± 50.8 b	543 ± 330 a	547 ± 300 a	4.53E-09	4.74E-08
Triacylglycerols	TG(18:3 38:6)	1.28 ± 0.44 a	0.156 ± 0.06 c	0.316 ± 0.137 b	4.88E-09	5.01E-08
Triacylglycerols	TG(20:5 34:2)	6.82 ± 2.7 a	1.38 ± 0.66 c	3.43 ± 0.85 b	5.36E-09	5.41E-08
Triacylglycerols	TG(18:3 30:0)	0.216 ± 0.078 c	0.394 ± 0.163 b	0.968 ± 0.343 a	5.86E-09	5.81E-08
PC	PC ae C34:3	0.426 ± 0.078 a	0.234 ± 0.034 c	0.291 ± 0.03 b	7.12E-09	6.94E-08
Triacylglycerols	TG(17:1 34:1)	0.798 ± 0.468 b	3.92 ± 2.15 a	4.97 ± 2.64 a	7.80E-09	7.48E-08
Cholesterol Esters	CE(20:3)	3.03 ± 1.68 b	14 ± 6.5 a	12.4 ± 4.7 a	8.03E-09	7.58E-08
Triacylglycerols	TG(18:2 36:3)	101 ± 28 a	22.8 ± 12.6 b	32.4 ± 17.7 b	8.97E-09	8.33E-08
Triacylglycerols	TG(18:1 36:6)	1.33 ± 0.42 a	0.402 ± 0.158 c	0.837 ± 0.256 b	9.85E-09	9.00E-08

Triacylglycerols	TG(22:5 34:1)	5.11 ± 2.25 c	12.7 ± 5.2 b	22.9 ± 7 a	1.05E-08	9.42E-08
LysoPC	lysoPC a C16:1	6.89 ± 1.47 c	14.3 ± 4.2 b	19.4 ± 5.8 a	1.12E-08	9.91E-08
Cholesterol Esters	CE(16:1)	18.8 ± 5.9 c	40.4 ± 16.1 b	59.5 ± 13.7 a	1.17E-08	1.01E-07
Triacylglycerols	TG(20:3 34:0)	0.367 ± 0.157 b	1.49 ± 0.79 a	2.02 ± 1.08 a	1.15E-08	1.01E-07
Triacylglycerols	TG(14:0 34:1)	2.58 ± 2.07 b	19.8 ± 16.5 a	23.9 ± 13.5 a	1.43E-08	1.21E-07
Triacylglycerols	TG(18:3 38:5)	0.994 ± 0.33 a	0.21 ± 0.074 c	0.399 ± 0.154 b	1.49E-08	1.25E-07
Triacylglycerols	TG(18:1 33:1)	2.08 ± 0.91 b	9.02 ± 5.25 a	10.7 ± 5.1 a	1.58E-08	1.30E-07
Triacylglycerols	TG(16:0 36:5)	15.5 ± 5 a	4.43 ± 1.77 c	7.83 ± 2.67 b	1.84E-08	1.50E-07
Triacylglycerols	TG(18:0 36:4)	10.1 ± 3.1 a	2.56 ± 1.48 b	2.94 ± 1.29 b	2.21E-08	1.75E-07
Triacylglycerols	TG(18:1 32:1)	18.2 ± 14.9 b	135 ± 99 a	170 ± 108 a	2.20E-08	1.75E-07
Triacylglycerols	TG(14:0 34:0)	0.604 ± 0.168 b	2.28 ± 1.82 a	2.67 ± 1.27 a	2.74E-08	2.11E-07
Triacylglycerols	TG(18:3 34:3)	2.48 ± 1.03 a	0.573 ± 0.194 c	1.18 ± 0.56 b	2.71E-08	2.11E-07
Triacylglycerols	TG(16:0 36:2)	58.6 ± 32.8 b	325 ± 210 a	326 ± 189 a	2.91E-08	2.20E-07
Triacylglycerols	TG(18:1 31:0)	<LOD b	2.32 ± 1 a	2.34 ± 0.74 a	2.93E-08	2.20E-07
Triacylglycerols	TG(18:2 38:5)	11.4 ± 3.5 a	2.9 ± 1.37 c	5.82 ± 1.68 b	3.20E-08	2.37E-07
Triacylglycerols	TG(17:1 32:1)	0.473 ± 0.179 b	1.05 ± 0.62 a	1.6 ± 0.83 a	3.63E-08	2.65E-07
Diacylglycerols	DG(18:2 20:4)	0.884 ± 0.236 a	0.207 ± 0.05 c	0.317 ± 0.1 b	3.77E-08	2.72E-07
Triacylglycerols	TG(14:0 32:2)	0.467 ± 0.199 c	1.18 ± 1.02 b	3.39 ± 1.59 a	3.88E-08	2.73E-07
Triacylglycerols	TG(18:2 34:3)	25.4 ± 9.4 a	6.69 ± 2.66 c	13.8 ± 5.9 b	3.88E-08	2.73E-07
Triacylglycerols	TG(18:1 36:0)	1.41 ± 0.03 b	3.53 ± 2.62 a	2.51 ± 1.49 a	4.08E-08	2.81E-07
Triacylglycerols	TG(20:4 36:3)	19.6 ± 7 a	4.75 ± 2.38 c	7.88 ± 2.86 b	4.68E-08	3.18E-07
Triacylglycerols	TG(16:1 33:1)	0.552 ± 0.235 b	1.48 ± 0.79 a	2.23 ± 1.21 a	4.93E-08	3.32E-07
Triacylglycerols	TG(14:0 35:2)	0.164 ± 0.019 c	0.32 ± 0.164 b	0.517 ± 0.215 a	5.39E-08	3.59E-07
Diacylglycerols	DG(18:1 18:1)	1.13 ± 0.49 b	4.3 ± 2.04 a	4.36 ± 2.25 a	5.50E-08	3.61E-07
Triacylglycerols	TG(20:1 34:1)	1.09 ± 0.54 b	5.7 ± 3.73 a	5.73 ± 3.64 a	6.10E-08	3.96E-07
PC	PC aa C32:2	2.13 ± 0.36 b	1.71 ± 0.45 c	3.66 ± 0.88 a	6.74E-08	4.33E-07
LysoPC	lysoPC a C18:1	40.5 ± 6 b	76.4 ± 12.1 a	70.9 ± 17.8 a	6.83E-08	4.34E-07
Triacylglycerols	TG(18:1 32:0)	14.8 ± 10.3 b	111 ± 86 a	89.6 ± 59.3 a	6.91E-08	4.35E-07
Triacylglycerols	TG(20:1 32:1)	0.279 ± 0.171 b	1.44 ± 0.94 a	2.09 ± 1.26 a	8.18E-08	5.09E-07
Triacylglycerols	TG(20:3 32:2)	0.428 ± 0.173 c	0.704 ± 0.211 b	1.45 ± 0.51 a	9.22E-08	5.68E-07
Triacylglycerols	TG(14:0 38:4)	0.307 ± 0.147 c	0.602 ± 0.222 b	1.3 ± 0.47 a	9.68E-08	5.90E-07

Triacylglycerols	TG(16:1 34:0)	1.78 ± 1.42 b	12 ± 9.3 a	14.2 ± 9.7 a	1.09E-07	6.59E-07
Triacylglycerols	TG(16:1 34:1)	16 ± 14.2 b	109 ± 84 a	134 ± 94 a	1.28E-07	7.65E-07
Triacylglycerols	TG(20:4 34:3)	4.78 ± 2.29 a	1.13 ± 0.48 c	2.75 ± 0.93 b	1.31E-07	7.72E-07
Triacylglycerols	TG(18:2 30:0)	2.24 ± 1.35 c	5.38 ± 2.89 b	11.5 ± 4.6 a	1.40E-07	8.17E-07
Triacylglycerols	TG(20:4 33:2)	0.68 ± 0.127 a	0.235 ± 0.109 c	0.449 ± 0.129 b	1.46E-07	8.48E-07
Triacylglycerols	TG(16:0 34:1)	34.5 ± 23.9 b	246 ± 191 a	203 ± 142 a	1.64E-07	9.40E-07
PC	PC aa C34:4	2.39 ± 0.52 b	2.29 ± 0.52 b	4.66 ± 1.13 a	1.73E-07	9.48E-07
PC	PC aa C36:1	12.8 ± 2.5 c	28.9 ± 6.5 a	22.4 ± 7.8 b	1.68E-07	9.48E-07
Triacylglycerols	TG(16:0 32:0)	6.75 ± 4.1 b	49.1 ± 40.6 a	30.8 ± 19.3 a	1.72E-07	9.48E-07
Triacylglycerols	TG(18:3 34:2)	17.4 ± 5.8 a	5.44 ± 1.91 c	9.6 ± 3.91 b	1.70E-07	9.48E-07
Triacylglycerols	TG(18:1 33:0)	0.624 ± 0.173 b	2.5 ± 1.6 a	2.47 ± 1.21 a	1.75E-07	9.49E-07
Triacylglycerols	TG(16:0 32:1)	8.27 ± 7.62 b	58.7 ± 51.5 a	70.1 ± 47.6 a	1.85E-07	9.95E-07
PC	PC ae C34:2	2.76 ± 0.7 a	1.44 ± 0.24 c	1.94 ± 0.27 b	1.96E-07	1.03E-06
Triacylglycerols	TG(20:1 30:1)	<LOD c	0.472 ± 0.199 b	0.636 ± 0.295 a	1.96E-07	1.03E-06
Triacylglycerols	TG(20:3 36:4)	2.45 ± 0.62 a	0.705 ± 0.286 c	1.27 ± 0.56 b	2.03E-07	1.06E-06
Triacylglycerols	TG(16:0 34:0)	4.57 ± 2.47 b	30.2 ± 24 a	21.4 ± 14.1 a	2.34E-07	1.21E-06
Triacylglycerols	TG(14:0 38:5)	0.444 ± 0.175 b	0.517 ± 0.17 b	1.33 ± 0.4 a	2.73E-07	1.39E-06
LysoPC	lysoPC a C14:0	26.5 ± 3.2 b	26.3 ± 5 b	41.4 ± 6 a	2.89E-07	1.45E-06
Triacylglycerols	TG(14:0 35:1)	<LOD c	0.386 ± 0.102 b	0.442 ± 0.136 a	2.90E-07	1.45E-06
Triacylglycerols	TG(18:0 32:1)	0.589 ± 0.368 b	3.08 ± 2.58 a	3.55 ± 2.06 a	3.05E-07	1.51E-06
Triacylglycerols	TG(14:0 34:2)	3.1 ± 2.33 c	11.2 ± 7.7 b	19.6 ± 10.4 a	3.41E-07	1.68E-06
Triacylglycerols	TG(22:6 32:1)	1.54 ± 1.19 c	2.54 ± 1.04 b	6.82 ± 1.88 a	3.65E-07	1.78E-06
Triacylglycerols	TG(18:2 38:4)	8.05 ± 2.19 a	2.57 ± 1.24 c	5.33 ± 1.71 b	4.29E-07	2.07E-06
Triacylglycerols	TG(22:5 32:0)	0.749 ± 0.407 c	1.71 ± 0.7 b	2.94 ± 1.15 a	4.45E-07	2.14E-06
LysoPC	lysoPC a C17:0	3.6 ± 0.66 a	2.13 ± 0.32 c	2.95 ± 0.52 b	4.76E-07	2.25E-06
Triacylglycerols	TG(17:1 36:4)	0.751 ± 0.193 a	0.239 ± 0.112 c	0.442 ± 0.182 b	4.75E-07	2.25E-06
Diacylglycerols	DG(16:0 18:1)	1.88 ± 0.73 b	6.22 ± 2.85 a	5.56 ± 2.88 a	5.27E-07	2.47E-06
Triacylglycerols	TG(16:0 38:2)	1.18 ± 0.5 b	5.17 ± 3.37 a	5.07 ± 3.17 a	6.68E-07	3.08E-06
Triacylglycerols	TG(16:1 32:0)	3.03 ± 2.86 b	19.1 ± 17.1 a	23.4 ± 17.1 a	7.41E-07	3.39E-06
PC	PC ae C38:2	1.05 ± 0.22 b	0.84 ± 0.147 c	1.42 ± 0.2 a	7.53E-07	3.39E-06
Triacylglycerols	TG(20:4 32:2)	1.39 ± 0.57 b	0.676 ± 0.246 c	2.07 ± 0.58 a	7.50E-07	3.39E-06

Fatty Acids	FA(18:2)	65.3 ± 13 a	44.6 b	47.4 ± 7.1 b	8.12E-07	3.60E-06
Triacylglycerols	TG(14:0 36:3)	5.54 ± 2.55 c	9.63 ± 4.41 b	18.8 ± 7.1 a	8.13E-07	3.60E-06
PC	PC aa C38:3	10.3 ± 1.9 b	20.9 ± 6 a	21.9 ± 7.9 a	1.09E-06	4.67E-06
Triacylglycerols	TG(14:0 34:3)	0.946 ± 0.687 c	1.8 ± 0.83 b	4.45 ± 2.25 a	1.08E-06	4.67E-06
Triacylglycerols	TG(16:1 36:1)	1.62 ± 1.17 b	7.61 ± 4.73 a	10.2 ± 7.9 a	1.09E-06	4.67E-06
Triacylglycerols	TG(16:1 36:2)	9.16 ± 7.09 b	39.1 ± 23.5 a	56.4 ± 43 a	1.09E-06	4.67E-06
Triacylglycerols	TG(17:0 34:1)	0.598 ± 0.152 b	2.23 ± 1.4 a	2.18 ± 1.11 a	1.07E-06	4.67E-06
Triacylglycerols	TG(16:0 35:2)	1.2 ± 0.53 b	3.71 ± 1.94 a	4.77 ± 2.63 a	1.11E-06	4.72E-06
Triacylglycerols	TG(16:0 32:2)	4.46 ± 3.36 b	19.5 ± 15.9 a	29.1 ± 18.7 a	1.20E-06	5.06E-06
PC	PC ae C34:1	2.31 ± 0.28 b	3.32 ± 0.42 a	3.4 ± 0.57 a	1.26E-06	5.26E-06
Triacylglycerols	TG(16:0 32:3)	0.602 ± 0.378 c	1.28 ± 0.53 b	2.44 ± 1.21 a	1.48E-06	6.14E-06
Triacylglycerols	TG(20:4 35:3)	0.276 ± 0.096 a	0.0888 ± 0.0467 c	0.143 ± 0.039 b	1.50E-06	6.19E-06
SM	SM C24:1	14.3 ± 1.6 b	19.5 ± 1.8 a	18.1 ± 2 a	1.54E-06	6.32E-06
Triacylglycerols	TG(18:1 36:4)	62.9 ± 17.9 a	20.7 ± 10.1 b	29.6 ± 15.6 b	1.62E-06	6.60E-06
Triacylglycerols	TG(18:1 32:2)	6.95 ± 4.25 c	19.4 ± 11 b	33.5 ± 19.4 a	1.66E-06	6.72E-06
Triacylglycerols	TG(18:2 34:2)	161 ± 44 a	62.5 ± 28.1 c	105 ± 39 b	1.70E-06	6.81E-06
Triacylglycerols	TG(16:0 33:1)	0.902 ± 0.367 b	3.78 ± 2.41 a	3.68 ± 1.85 a	1.75E-06	6.96E-06
Triacylglycerols	TG(16:0 38:7)	2.46 ± 0.85 a	0.991 ± 0.359 b	2.15 ± 0.6 a	1.84E-06	7.27E-06
Cholesterol Esters	CE(22:5)	2.01 ± 0.69 b	4.69 ± 1.65 a	4.91 ± 1.76 a	2.08E-06	8.17E-06
PC	PC ae C32:1	0.629 ± 0.078 c	0.782 ± 0.069 b	0.928 ± 0.167 a	2.22E-06	8.64E-06
Triacylglycerols	TG(16:1 32:1)	4.36 ± 3.81 b	26.3 ± 25.1 a	39.1 ± 28.6 a	2.26E-06	8.76E-06
Triacylglycerols	TG(16:1 36:5)	1.43 ± 0.67 a	0.469 ± 0.165 b	1.18 ± 0.51 a	2.66E-06	1.02E-05
Triacylglycerols	TG(18:1 38:7)	1.89 ± 0.72 a	0.689 ± 0.248 b	1.4 ± 0.47 a	3.10E-06	1.19E-05
Triacylglycerols	TG(14:0 36:4)	3.59 ± 1.14 a	1.94 ± 0.79 b	4.74 ± 1.51 a	3.41E-06	1.30E-05
PC	PC ae C36:2	3.4 ± 0.81 a	2.06 ± 0.33 b	3.25 ± 0.57 a	3.52E-06	1.33E-05
Triacylglycerols	TG(20:4 32:1)	2.66 ± 1.75 c	3.67 ± 1.18 b	9.08 ± 3.55 a	3.59E-06	1.35E-05
Triacylglycerols	TG(18:2 35:2)	1.74 ± 0.41 a	0.688 ± 0.371 c	1.23 ± 0.5 b	3.76E-06	1.40E-05
Triacylglycerols	TG(20:4 34:2)	35.7 ± 12.5 a	12.2 ± 5.9 c	24.5 ± 8.2 b	3.77E-06	1.40E-05
Triacylglycerols	TG(16:0 36:4)	102 ± 28 a	42 ± 19.7 c	64.3 ± 21.3 b	4.29E-06	1.58E-05
Triacylglycerols	TG(22:6 34:3)	2.35 ± 1.21 a	0.673 ± 0.354 b	1.88 ± 0.55 a	4.72E-06	1.72E-05
Triacylglycerols	TG(16:1 32:2)	1.13 ± 0.91 c	3.67 ± 2.78 b	7.23 ± 4.98 a	5.04E-06	1.83E-05

Diacylglycerols	DG(16:0 16:1)	0.414 ± 0.202 b	1.12 ± 0.58 a	1.39 ± 0.72 a	5.27E-06	1.90E-05
Triacylglycerols	TG(16:0 28:2)	0.184 ± 0.062 c	0.377 ± 0.195 b	0.674 ± 0.313 a	5.31E-06	1.91E-05
Triacylglycerols	TG(20:2 34:1)	1.64 ± 0.79 b	4.43 ± 2.47 a	5.77 ± 2.86 a	5.75E-06	2.05E-05
Triacylglycerols	TG(18:1 36:1)	8.74 ± 4.8 b	39.4 ± 28.2 a	36.2 ± 27.9 a	6.32E-06	2.24E-05
Diacylglycerols	DG(18:2 18:3)	1.16 ± 0.24 a	0.329 ± 0.063 b	0.363 ± 0.089 b	6.45E-06	2.26E-05
Triacylglycerols	TG(16:0 35:1)	0.63 ± 0.149 b	2.32 ± 1.43 a	2.19 ± 1.14 a	6.44E-06	2.26E-05
PC	PC ae C36:1	0.922 ± 0.147 c	1.19 ± 0.15 b	1.41 ± 0.25 a	7.69E-06	2.68E-05
Triacylglycerols	TG(20:2 32:1)	0.452 ± 0.306 c	1.21 ± 0.81 b	1.92 ± 0.95 a	9.24E-06	3.20E-05
Triacylglycerols	TG(16:0 36:6)	1.16 ± 0.53 a	0.472 ± 0.151 b	0.94 ± 0.301 a	9.61E-06	3.31E-05
Triacylglycerols	TG(18:0 38:6)	1.34 ± 0.42 a	0.502 ± 0.242 c	0.7 ± 0.273 b	9.94E-06	3.40E-05
PC	PC aa C40:5	6.59 ± 1.33 b	12.6 ± 2.8 a	13.4 ± 6 a	1.15E-05	3.90E-05
Triacylglycerols	TG(18:1 36:2)	40 ± 19.9 b	160 ± 110 a	168 ± 136 a	1.23E-05	4.15E-05
Triacylglycerols	TG(22:5 34:3)	1.42 ± 0.57 a	0.739 ± 0.277 b	1.93 ± 0.49 a	1.38E-05	4.64E-05
Triacylglycerols	TG(22:6 34:2)	18 ± 7.6 a	6.95 ± 3.43 b	17.3 ± 4.6 a	1.49E-05	4.99E-05
Triacylglycerols	TG(16:0 38:1)	0.3 ± 0.123 b	1.26 ± 0.92 a	0.918 ± 0.584 a	1.68E-05	5.60E-05
Triacylglycerols	TG(20:0 32:4)	0.82 ± 0.266 a	0.349 ± 0.124 c	0.545 ± 0.196 b	1.75E-05	5.79E-05
Triacylglycerols	TG(16:0 40:8)	4.21 ± 1.75 a	1.57 ± 0.75 b	3.51 ± 1 a	1.79E-05	5.88E-05
SM	SM C18:0	1.7 ± 0.3 c	2.67 ± 0.32 a	2.29 ± 0.57 b	1.81E-05	5.91E-05
Triacylglycerols	TG(22:6 34:1)	9.84 ± 5.2 b	13.1 ± 6.1 b	26.2 ± 6 a	2.14E-05	6.95E-05
SM	SM C20:2	0.076 ± 0.0275 a	0.0529 ± 0.0276 b	0.0253 ± 0.0125 c	2.44E-05	7.88E-05
Triacylglycerols	TG(16:0 34:2)	57.7 ± 38.3 b	182 ± 112 a	212 ± 132 a	2.64E-05	8.49E-05
Triacylglycerols	TG(16:1 36:4)	9.39 ± 4.1 a	3.74 ± 1.45 b	8.34 ± 3.83 a	2.87E-05	9.18E-05
Triacylglycerols	TG(18:2 32:1)	19.2 ± 12.4 c	34 ± 14.2 b	62.2 ± 31.3 a	3.09E-05	9.77E-05
Triacylglycerols	TG(18:0 32:2)	0.516 ± 0.274 c	1.09 ± 0.75 b	1.75 ± 0.77 a	3.12E-05	9.83E-05
Triacylglycerols	TG(20:1 34:0)	0.194 ± 0.077 b	0.678 ± 0.449 a	0.584 ± 0.344 a	3.15E-05	9.84E-05
Triacylglycerols	TG(18:2 32:2)	7.43 ± 2.75 b	4.38 ± 1.62 c	10.6 ± 4.4 a	3.28E-05	1.02E-04
Triacylglycerols	TG(20:2 32:0)	0.24 ± 0.122 b	0.641 ± 0.335 a	0.73 ± 0.392 a	3.63E-05	1.12E-04
PC	PC ae C40:4	1.69 ± 0.21 b	1.48 ± 0.19 b	2.23 ± 0.45 a	3.71E-05	1.14E-04
PC	PC aa C36:6	0.52 ± 0.128 b	0.47 ± 0.116 b	0.823 ± 0.21 a	4.31E-05	1.31E-04
PC	PC ae C34:0	0.554 ± 0.087 b	0.511 ± 0.051 b	0.718 ± 0.117 a	4.31E-05	1.31E-04
Triacylglycerols	TG(16:0 38:6)	9.42 ± 3.55 a	3.99 ± 1.74 b	7.71 ± 2.4 a	4.33E-05	1.31E-04

PC	PC aa C42:4	0.18 ± 0.021 a	0.126 ± 0.014 b	0.162 ± 0.03 a	4.47E-05	1.35E-04
Triacylglycerols	TG(16:1 34:2)	19 ± 15.2 b	45.7 ± 23.4 a	76.6 ± 50.4 a	4.58E-05	1.37E-04
Triacylglycerols	TG(22:6 32:0)	1.4 ± 0.83 c	2.44 ± 1.01 b	3.77 ± 1.54 a	5.05E-05	1.51E-04
Triacylglycerols	TG(16:1 38:4)	0.994 ± 0.626 b	1.3 ± 0.4 b	2.98 ± 1.61 a	5.52E-05	1.64E-04
Cholesterol Esters	CE(17:1)	0.988 ± 0.229 c	1.32 ± 0.42 b	1.84 ± 0.48 a	5.96E-05	1.76E-04
Triacylglycerols	TG(20:3 34:2)	4.69 ± 1.98 c	6.75 ± 2.3 b	11.5 ± 4.7 a	6.55E-05	1.93E-04
Triacylglycerols	TG(18:0 32:0)	1.19 ± 0.32 b	4.76 ± 3.92 a	2.51 ± 1.19 a	7.39E-05	2.15E-04
Triacylglycerols	TG(22:5 34:2)	8.95 ± 3.22 b	7 ± 3.06 b	16 ± 4.1 a	7.69E-05	2.23E-04
PC	PC ae C38:3	0.727 ± 0.159 c	0.851 ± 0.125 b	1.09 ± 0.19 a	7.79E-05	2.24E-04
PC	PC ae C38:4	6.36 ± 0.95 a	4.46 ± 0.66 b	5.81 ± 1.01 a	9.48E-05	2.71E-04
Triacylglycerols	TG(20:1 32:2)	0.332 ± 0.097 c	0.548 ± 0.258 b	1.03 ± 0.53 a	1.01E-04	2.87E-04
Triacylglycerols	TG(18:3 35:2)	0.304 ± 0.106 a	0.104 ± 0.042 c	0.187 ± 0.097 b	1.08E-04	3.04E-04
Triacylglycerols	TG(17:1 34:2)	0.996 ± 0.451 c	1.6 ± 0.67 b	2.68 ± 1.33 a	1.08E-04	3.05E-04
Triacylglycerols	TG(22:4 32:0)	0.474 ± 0.258 c	0.795 ± 0.311 b	1.42 ± 0.6 a	1.13E-04	3.14E-04
Triacylglycerols	TG(18:1 35:2)	1.22 ± 0.43 b	2.46 ± 1.28 a	3.47 ± 2.06 a	1.15E-04	3.20E-04
PC	PC ae C38:1	0.141 ± 0.053 b	0.25 ± 0.11 a	0.329 ± 0.101 a	1.33E-04	3.66E-04
Triacylglycerols	TG(20:5 36:2)	2.33 ± 1.09 a	0.938 ± 0.36 b	1.71 ± 0.66 a	1.37E-04	3.77E-04
Acylcarnitines	C12	<LOD b	0.0747 ± 0.0012 b	0.0809 ± 0.007 a	1.40E-04	3.83E-04
Triacylglycerols	TG(22:4 34:2)	6.88 ± 2.55 a	3.15 ± 1.36 b	6.71 ± 2.16 a	1.65E-04	4.48E-04
Triacylglycerols	TG(16:0 38:4)	4.4 ± 2.07 c	6.86 ± 2.6 b	11 ± 5 a	1.66E-04	4.50E-04
Triacylglycerols	TG(18:1 34:2)	125 ± 57 b	242 ± 119 a	325 ± 171 a	1.74E-04	4.69E-04
Triacylglycerols	TG(18:3 32:1)	1.57 ± 1.02 b	2.19 ± 0.84 b	4.3 ± 2.18 a	1.94E-04	5.22E-04
Triacylglycerols	TG(16:0 38:3)	3.19 ± 1.34 b	6.07 ± 3.02 a	8.32 ± 4.12 a	2.05E-04	5.46E-04
Triacylglycerols	TG(18:1 32:3)	0.782 ± 0.365 b	1.13 ± 0.5 b	2.1 ± 1.14 a	2.12E-04	5.63E-04
Triacylglycerols	TG(22:4 32:2)	0.363 ± 0.153 b	0.232 ± 0.064 c	0.564 ± 0.185 a	2.54E-04	6.72E-04
Fatty Acids	DHA	14 ± 4.8 a	7.29 ± 2.14 c	9.64 ± 1.95 b	2.76E-04	7.26E-04
PC	PC ae C40:6	1.56 ± 0.21 a	1.09 ± 0.14 b	1.39 ± 0.29 a	2.82E-04	7.40E-04
Triacylglycerols	TG(16:0 34:3)	20 ± 14.7 b	33.4 ± 13.4 a	57.9 ± 33.6 a	2.95E-04	7.71E-04
Triacylglycerols	TG(18:1 38:6)	7.2 ± 2.84 a	3.89 ± 1.67 b	7.55 ± 2.26 a	3.34E-04	8.68E-04
PC	PC ae C40:3	0.314 ± 0.058 b	0.394 ± 0.065 a	0.466 ± 0.1 a	3.53E-04	9.15E-04
Triacylglycerols	TG(16:1 38:5)	1.49 ± 0.8 b	1.32 ± 0.45 b	3.23 ± 1.52 a	3.95E-04	1.02E-03

Triacylglycerols	TG(20:5 34:0)	0.649 ± 0.258 b	0.451 ± 0.163 c	0.856 ± 0.216 a	4.09E-04	1.05E-03
PC	PC aa C40:2	0.247 ± 0.035 a	0.175 ± 0.036 c	0.207 ± 0.035 b	5.09E-04	1.29E-03
Triacylglycerols	TG(16:0 40:7)	6.44 ± 2.5 b	4.81 ± 2.33 b	9.75 ± 2.34 a	5.46E-04	1.38E-03
Cholesterol Esters	CE(15:0)	3.37 ± 0.81 b	2.78 ± 0.84 b	4.28 ± 0.74 a	5.82E-04	1.47E-03
Triacylglycerols	TG(16:0 40:6)	6.55 ± 2.44 b	5.22 ± 2.1 b	10.5 ± 2.7 a	5.89E-04	1.48E-03
Triacylglycerols	TG(16:1 38:3)	0.619 ± 0.32 b	0.964 ± 0.428 b	1.86 ± 1.24 a	6.12E-04	1.53E-03
Triacylglycerols	TG(20:5 34:1)	4.06 ± 1.68 a	2.66 ± 1.02 b	5.17 ± 1.29 a	6.65E-04	1.66E-03
PC	PC aa C40:3	0.248 ± 0.051 b	0.347 ± 0.068 a	0.351 ± 0.072 a	6.91E-04	1.71E-03
Triacylglycerols	TG(18:0 38:7)	0.496 ± 0.164 a	0.246 ± 0.094 b	0.317 ± 0.101 b	7.84E-04	1.94E-03
Triacylglycerols	TG(16:0 37:3)	0.175 ± 0.069 b	0.245 ± 0.112 b	0.367 ± 0.135 a	8.18E-04	2.01E-03
PC	PC aa C34:3	6.47 ± 0.82 a	4.7 ± 1.05 b	6.92 ± 2.06 a	8.31E-04	2.03E-03
Aminoacids Related	Betaine	69.9 ± 16.4 a	49.9 ± 9.6 b	50.3 ± 8.9 b	1.01E-03	2.45E-03
Ceramides	Cer(d18:1/24:1)	0.697 ± 0.192 b	1.09 ± 0.18 a	1.12 ± 0.47 a	1.22E-03	2.95E-03
Triacylglycerols	TG(17:0 34:3)	0.237 ± 0.081 b	0.263 ± 0.091 b	0.507 ± 0.268 a	1.23E-03	2.96E-03
Triacylglycerols	TG(17:2 36:3)	0.405 ± 0.09 a	0.211 ± 0.052 b	0.252 ± 0.106 b	1.24E-03	2.97E-03
Triacylglycerols	TG(16:0 35:3)	0.829 ± 0.373 b	1.12 ± 0.49 b	1.76 ± 0.77 a	1.26E-03	3.00E-03
Cholesterol Esters	CE(17:0)	2.07 ± 0.68 a	1.56 ± 0.52 b	2.52 ± 0.49 a	1.47E-03	3.48E-03
Triacylglycerols	TG(16:1 34:3)	3.84 ± 3.3 b	5.09 ± 2.16 b	11.1 ± 7.7 a	1.54E-03	3.66E-03
PC	PC ae C38:6	1.69 ± 0.26 a	1.27 ± 0.16 b	1.51 ± 0.26 a	1.56E-03	3.67E-03
Triacylglycerols	TG(20:3 34:3)	1.26 ± 0.57 b	0.917 ± 0.289 b	1.92 ± 0.86 a	1.72E-03	4.03E-03
Triacylglycerols	TG(20:2 34:3)	0.688 ± 0.309 a	0.372 ± 0.117 b	0.835 ± 0.451 a	1.80E-03	4.20E-03
Triacylglycerols	TG(16:0 34:4)	1.57 ± 0.99 b	1.73 ± 0.6 b	3.47 ± 1.92 a	1.82E-03	4.23E-03
Triacylglycerols	TG(16:0 38:5)	7.59 ± 3.23 b	7.71 ± 3.19 b	13.8 ± 5 a	1.92E-03	4.44E-03
Triacylglycerols	TG(20:1 34:2)	1.78 ± 0.76 b	2.98 ± 1.52 a	4.24 ± 2.51 a	1.93E-03	4.44E-03
Triacylglycerols	TG(18:1 33:2)	1.81 ± 0.48 b	2.31 ± 1.14 b	3.35 ± 1.31 a	2.37E-03	5.43E-03
HexCer	HexCer(d18:1/24:1)	0.96 ± 0.292 b	1.39 ± 0.26 a	1.34 ± 0.36 a	2.49E-03	5.69E-03
Triacylglycerols	TG(16:0 36:3)	130 ± 55 b	192 ± 94 a	254 ± 105 a	2.61E-03	5.94E-03
SM	SM C26:1	0.219 ± 0.035 b	0.263 ± 0.046 a	0.297 ± 0.049 a	2.62E-03	5.94E-03
Triacylglycerols	TG(20:4 32:0)	3.21 ± 1.53 b	5.39 ± 2.91 a	6.39 ± 2.67 a	2.95E-03	6.67E-03
Triacylglycerols	TG(16:1 36:3)	15.5 ± 9.1 b	18.7 ± 8 b	36 ± 23.2 a	3.21E-03	7.18E-03
Triacylglycerols	TG(18:0 36:1)	0.79 ± 0.28 b	2.19 ± 1.72 a	1.47 ± 1.00 a	3.27E-03	7.30E-03

Fatty Acids	AA	29.8 ± 8.1 a	20.1 ± 6.6 b	21.7 ± 3.6 b	3.49E-03	7.73E-03
SM	SM C18:1	0.762 ± 0.096 b	0.945 ± 0.099 a	0.892 ± 0.145 a	3.66E-03	8.08E-03
Triacylglycerols	TG(18:3 36:2)	7.87 ± 2.75 a	3.89 ± 1.95 b	5.19 ± 3.39 b	3.89E-03	8.55E-03
Diacylglycerols	DG(16:1 18:2)	0.9 ± 0.508 b	0.963 ± 0.221 b	1.68 ± 0.81 a	3.91E-03	8.57E-03
PC	PC aa C34:2	154 ± 26 a	118 ± 16 b	140 ± 25 a	3.98E-03	8.66E-03
Triacylglycerols	TG(17:0 34:2)	0.919 ± 0.33 b	1.41 ± 0.71 a	1.88 ± 0.93 a	3.98E-03	8.66E-03
Cholesterol Esters	CE(20:5)	12.3 ± 2.3 b	14.8 ± 5.1 b	18 ± 3.4 a	4.19E-03	9.07E-03
Triacylglycerols	TG(20:4 34:1)	19.4 ± 8.1 b	21.5 ± 9.5 b	35.4 ± 13.6 a	4.44E-03	9.58E-03
Triacylglycerols	TG(18:1 38:5)	6.68 ± 2.48 b	6.46 ± 2.58 b	11.6 ± 4.8 a	4.85E-03	1.04E-02
Diacylglycerols	DG(18:1 18:3)	0.505 ± 0.109 a	0.361 ± 0.098 b	0.379 ± 0.095 b	4.98E-03	1.07E-02
Diacylglycerols	DG(16:1 18:1)	2.22 ± 1.28 b	3.07 ± 1.53 a	3.42 ± 1.72 a	5.15E-03	1.10E-02
Cholesterol Esters	CE(20:0)	0.839 ± 0.204 a	0.735 ± 0.136 b	0.747 ± 0.173 a	5.35E-03	1.14E-02
Triacylglycerols	TG(20:4 34:0)	2.8 ± 1.17 b	3.77 ± 1.78 ab	5.19 ± 2.02 a	5.72E-03	1.21E-02
Triacylglycerols	TG(20:0 32:3)	0.413 ± 0.122 b	0.523 ± 0.202 b	0.718 ± 0.279 a	5.84E-03	1.23E-02
Triacylglycerols	TG(18:2 34:1)	118 ± 48 b	170 ± 85 ab	219 ± 93 a	5.87E-03	1.24E-02
Indoles Derivatives	3-IPA	0.967 ± 0.358 a	0.62 ± 0.236 b	0.574 ± 0.136 b	5.98E-03	1.25E-02
Diacylglycerols	DG(18:1 20:3)	0.121 ± 0.037 b	0.168 ± 0.033 a	0.208 ± 0.084 a	6.40E-03	1.34E-02
Triacylglycerols	TG(18:3 36:1)	1.91 ± 0.62 a	1.09 ± 0.53 b	1.18 ± 0.61 b	6.77E-03	1.41E-02
PC	PC aa C38:5	38.8 ± 6.4 b	44.9 ± 8.2 ab	56.5 ± 17.4 a	7.19E-03	1.49E-02
SM	SM (OH) C24:1	0.967 ± 0.123 b	0.982 ± 0.108 b	1.16 ± 0.16 a	7.62E-03	1.58E-02
Triacylglycerols	TG(18:0 36:2)	4.07 ± 1.93 b	10.4 ± 8 a	7.75 ± 5.49 a	7.82E-03	1.61E-02
Triacylglycerols	TG(17:2 38:7)	0.0678 ± 0.0173 a	0.0643 ± 0.0262 b	0.059 ± 0.0104 b	8.52E-03	1.75E-02
Triacylglycerols	TG(17:1 34:3)	0.284 ± 0.102 b	0.255 ± 0.078 b	0.543 ± 0.291 a	9.00E-03	1.84E-02
Triacylglycerols	TG(16:0 33:2)	1.11 ± 0.38 b	1.77 ± 0.93 a	2.24 ± 1.12 a	9.05E-03	1.84E-02
Triacylglycerols	TG(17:2 34:2)	0.377 ± 0.113 a	0.242 ± 0.08 b	0.357 ± 0.114 a	9.49E-03	1.93E-02
Triacylglycerols	TG(18:1 35:3)	0.808 ± 0.291 b	0.794 ± 0.294 b	1.33 ± 0.67 a	9.66E-03	1.96E-02
Triacylglycerols	TG(18:2 32:0)	22.5 ± 11.4 b	39 ± 21.3 a	43.4 ± 22.5 a	9.72E-03	1.96E-02
Cholesterol Esters	CE(15:1)	0.536 ± 0.118 b	0.551 ± 0.163 b	0.709 ± 0.119 a	1.13E-02	2.26E-02
Diacylglycerols	DG(18:1 20:2)	0.147 ± 0.035 b	0.149 ± 0.026 a	0.186 ± 0.054 a	1.20E-02	2.37E-02
Triacylglycerols	TG(18:1 33:3)	0.326 ± 0.097 ab	0.257 ± 0.102 b	0.416 ± 0.162 a	1.28E-02	2.50E-02
LysoPC	lysoPC a C26:1	0.777 ± 0.101 a	0.967 ± 0.361 a	0.895 ± 0.228 b	1.30E-02	2.55E-02

Diacylglycerols	DG(14:0 18:1)	<LOD b	0.916 ± 0.131 ab	0.904 ± 0.304 a	1.34E-02	2.61E-02
SM	SM (OH) C22:1	5.03 ± 0.43 a	4.29 ± 0.47 b	4.66 ± 0.62 ab	1.45E-02	2.82E-02
Triacylglycerols	TG(18:2 33:1)	2.5 ± 0.79 b	2.5 ± 1.16 b	3.75 ± 1.3 a	1.47E-02	2.85E-02
Triacylglycerols	TG(17:1 36:3)	0.869 ± 0.342 b	0.755 ± 0.328 b	1.35 ± 0.69 a	1.51E-02	2.92E-02
PC	PC aa C36:5	5.95 ± 1.1 ab	5.09 ± 1.1 b	7.09 ± 2 a	1.52E-02	2.93E-02
Fatty Acids	FA(20:3)	1.47 ± 0.38 a	0.968 ± 0.354 b	1.34 ± 0.44 a	1.56E-02	3.00E-02
LysoPC	lysoPC a C18:2	111 ± 21 a	86.1 ± 14.7 b	96.1 ± 18.7 ab	1.59E-02	3.03E-02
PC	PC aa C36:3	33 ± 5.9 b	38.4 ± 8.3 ab	44.9 ± 11.9 a	1.68E-02	3.20E-02
Triacylglycerols	TG(18:1 34:4)	2.09 ± 1.08 ab	1.54 ± 0.53 b	3.02 ± 1.7 a	1.70E-02	3.21E-02
Cholesterol Esters	CE(18:2)	212 ± 59 a	148 ± 55 b	174 ± 44 ab	1.77E-02	3.35E-02
Triacylglycerols	TG(20:1 34:3)	0.526 ± 0.258 b	0.504 ± 0.235 b	0.995 ± 0.634 a	1.84E-02	3.45E-02
Triacylglycerols	TG(18:1 34:3)	24.4 ± 13.2 b	26.9 ± 11.7 b	46.6 ± 28.9 a	1.90E-02	3.55E-02
Triacylglycerols	TG(20:1 32:3)	0.213 ± 0.072 a	0.176 ± 0.048 b	0.271 ± 0.124 a	1.90E-02	3.55E-02
Ceramides	Cer(d18:1/22:0)	0.591 ± 0.169 a	0.468 ± 0.073 ab	0.416 ± 0.188 b	2.18E-02	4.04E-02
PC	PC ae C40:5	1.11 ± 0.17 ab	1.03 ± 0.14 b	1.27 ± 0.21 a	2.23E-02	4.11E-02
Cholesterol Esters	CE(18:1)	51.8 ± 14.6 b	74.4 ± 28.5 a	67.4 ± 17.4 a	2.32E-02	4.27E-02
Triacylglycerols	TG(20:4 36:2)	11.3 ± 4.3 a	7.11 ± 3.3 b	11.1 ± 5.3 a	2.38E-02	4.37E-02
Aminoacids Related	Ornithine	67.1 ± 10 a	54.6 ± 6.2 b	65.4 ± 14.5 a	2.55E-02	4.65E-02
HexCer	HexCer(d18:1/22:0)	1.07 ± 0.26 a	0.935 ± 0.207 ab	0.788 ± 0.2 b	2.59E-02	4.73E-02

Data are means ± SD for n = 10 rats/group. Means not sharing the same lowercase letter (a, b) differ across groups, $P < 0.05$.

Abbreviations: LOD, limit of detection.
